# Efficacy of dexamethasone suppression test during the diagnosis of primary pigmented nodular adrenocortical disease in Chinese adrenocorticotropic hormone-independent Cushing syndrome

**DOI:** 10.1007/s12020-017-1436-9

**Published:** 2017-11-01

**Authors:** Shi Chen, Ran Li, Lin Lu, Lian Duan, Xuebin Zhang, Anli Tong, Hui Pan, Huijuan Zhu, Zhaolin Lu

**Affiliations:** 1 Department of Endocrinology, Peking Union Medical College Hospital, Peking Union Medical College, Chinese Academy of Medical Sciences, Key Laboratory of Endocrinology of National Health and Family Planning Commission, Beijing, China; 2 Eight-year Program of Clinical Medicine, Peking Union Medical College Hospital, Peking Union Medical College, Chinese Academy of Medical Sciences, Beijing, China; 3 Department of Urology, Peking Union Medical College Hospital, Peking Union Medical College, Chinese Academy of Medical Sciences, Beijing, China

**Keywords:** Cushing syndrome, Adrenal gland diseases, Carney complex, Primary pigmented nodular adrenocortical disease, Dexamethasone suppression test

## Abstract

**Objective:**

To evaluate the cut-off value of the ratio of 24 h urinary free cortisol (24 h UFC) levels post-dexamethasone to prior-dexamethasone in dexamethasone suppression test (DST) during the diagnosis of primary pigmented nodular adrenocortical disease in Chinese adrenocorticotropic hormone-independent Cushing syndrome.

**Design:**

Retrospective study.

**Participants:**

The patients diagnosed with primary pigmented nodular adrenocortical disease (PPNAD, *n* = 25), bilateral macronodular adrenal hyperplasia (BMAH, *n* = 27), and adrenocortical adenoma (ADA, *n* = 84) were admitted to the Peking Union Medical College Hospital from 2001 to 2016.

**Estimations:**

Serum cortisol, adrenocorticotropic hormone (ACTH), and 24 h UFC were measured before and after low-dose dexamethasone suppression test (LDDST) and high-dose dexamethasone suppression test (HDDST).

**Results:**

After LDDST and HDDST, 24 h UFC elevated in patients with PPNAD (paired *t*-test, *P* = 0.007 and *P* = 0.001), while it remained unchanged in the BMAH group (paired *t*-test, *P* = 0.471 and *P* = 0.414) and decreased in the ADA group (paired *t*-test, *P* = 0.002 and *P* = 0.004). The 24 h UFC level after LDDST was higher in PPNAD and BMAH as compared to ADA (*P* < 0.017), while no significant difference was observed between PPNAD and BMAH. After HDDST, 24 h UFC was higher in patients with PPNAD as compared to that of ADA and BMAH (*P* < 0.017). The cut-off value of 24 h UFC (Post-L-Dex)/(Pre-L-Dex) was 1.16 with 64.0% sensitivity and 77.9% specificity, and the cut-off value of 24 h UFC (Post-H-Dex)/(Pre-H-Dex) was 1.08 with 84.0% sensitivity and 75.6% specificity.

**Conclusion:**

The ratio of post-dexamethasone to prior-dexamethasone had a unique advantage in distinguishing PPNAD from BMAH and ADA.

## Introduction

Cushing syndrome (CS) is characterized by chronic exposure to excessive glucocorticoid, and thus classified into adrenocorticotropic hormone (ACTH)-dependent and ACTH-independent CS. ACTH-independent CS accounts for approximately 15–20% of CS, primarily due to the unilateral adrenal tumor such as adrenocortical adenoma (ADA) and adrenal carcinoma that accounts for 10 and 5%, respectively. The rare causes of ACTH-independent CS include primary bilateral macronodular adrenal hyperplasia (BMAH), primary pigmented nodular adrenal disease (PPNAD), and McCune–Albright syndrome [1].

PPNAD is rarely encountered and accounts for 0.6–1.9% of CS [2]. It can occur isolated or as a component of the Carney complex (CNC). CNC is a hereditary multiple neoplasia syndrome characterized by skin lentigines, myxomas, and endocrine tumors, first described by J. Aidan Carney in 1985 [3]. According to Stratakis et al., 95% patients with PPNAD fulfilled the diagnostic criteria of CNC [4]; however, other reports on the incidence rate of CNC in patients with PPNAD are limited. The pathological feature of PPNAD constitutes of multiple pigmented cortical nodules and atrophy of the internodular cortex. The typical radiological features of PPNAD may present as multiple nodules of bilateral adrenal glands; however, in most cases, the appearance of adrenal glands with PPNAD might be normal or presented with macronodules [5] that are indistinguishable from BMAH, which is characterized by large adrenal nodules, >5 cm. Thus, occasionally, the preoperative diagnosis through imaging is misleading and rather challenging [5, 6]. Therefore, a new method with high sensitivity and specificity that can discriminate PPNAD from other adrenal diseases is an urgent prerequisite.

Several reports have mentioned an anomalous increase in the glucocorticoid level in patients with PPNAD [7–9]. Stratakis et al. reported a paradoxical increase in urinary free cortisol (UFC) in response to dexamethasone during the Liddle test. Moreover, an increase of more than 50% in 24 h UFC on day 6 could distinguish PPNAD patients from those with other primary adrenal disorders causing CS with a 69.2% sensitivity and 80.0% specificity [4]. Dexamethasone suppression tests (DST) are widely used in the diagnosis of CS. Low-dose dexamethasone suppression test (LDDST) serves as the definitive test in the confirmation of CS, while the high-dose dexamethasone suppression test (HDDST) differentiates between pituitary-dependent and non-pituitary-dependent forms of CS. Nevertheless, the usefulness of DST in the diagnosis of PPNAD is yet to be elucidated. In addition, since the level of cortisol differs among races, whether an increase in >50% of 24 h UFC in the Liddle test could also be applied to Asian people is yet to be investigated.

Herein, we analyzed data from 25 patients with PPNAD and control groups consisting of 27 patients with BMAH and 84 with ADA were admitted to the Peking Union Medical College Hospital (PUMCH) from 2001 to 2016. To our knowledge, this was the largest single-center study of PPNAD in Asia. LDDST and HDDST were conducted, and comparisons were made among the three groups. We used the ratio of 24 h UFC post-dexamethasone to prior-dexamethasone, for the first time, to determine the cut-off value for the evaluation of PPNAD.

## Materials and methods

### Patients

The present study was approved by the Ethics Committee of PUMCH. We retrospectively analyzed the records of 136 patients with ACTH-independent CS who were admitted to PUMCH from 2001 to 2016. All of the patients underwent unilateral epinephrectomy for the preserving of the adrenal gland function. Twenty-five patients were histologically confirmed for PPNAD, 27 patients had BMAH, and 84 were diagnosed with ADA.

### Methods

All the clinical information was extracted from the medical records of PUMCH. The CNC was diagnosed based on the published criteria in 2001 [3]. According to the criteria, the CNC could be diagnosed if patients fulfilled two major criteria or one major and one supplementary criterion. The screening tests, including echocardiogram, thyroid ultrasonography, breast ultrasonography, pituitary MRI, testicular ultrasonography in males, and transabdominal pelvic ultrasonography in females, were conducted for the confirmation of CNC in all participants. A total of 109 patients received LDDST, and 27 patients were administered 1 mg overnight dexamethasone test. All patients presented abnormal screening test results; moreover, they undertook HDDST immediately after LDDST. LDDST: After 2 days of baseline measurement of 24 h UFC, dexamethasone was administered orally at the dosage of 0.5 mg every 6 h for 2 days at 8:00, 14:00, 20:00, and 8:00. The 24 h UFC was measured on the second day of dexamethasone administration. The ratio of 24 h UFC post-LDDST to before LDDST was denoted as 24 h UFC (Post-L-Dex)/(Pre-L-Dex). Similar to LDDST, dexamethasone was administered at the dosage of 2 mg during HDDST. The time point of 24 h UFC was identical to that of LDDST. The ratio of 24 h UFC after HDDST to before HDDST was denoted as 24 h UFC (Post-H-Dex)/(Pre-H-Dex). Serum cortisol, serum ACTH, and 24 h UFC were measured with the chemiluminescence method using the commercial kits (DPC Biotechnology and Medical Products Cooperation, Tianjin, China). The data of arterial blood pressure, serum sodium and potassium, fasting plasma glucose, plasma glucose after the 75 g oral glucose tolerance test, and HbA1c levels were collected. Computed tomography (CT) of adrenal glands was performed for each patient, and bone mineral density (BMD) was evaluated using Dual-Energy X-ray absorption assay method to assess the influence of hypercortisolism to bone metabolism.

### Statistical analysis

Statistical analyses were performed using the SPSS statistical package version 22.0. Kolmogorov–Smirnov test was utilized for normal distribution and homogeneity test for a variance. Normal distribution data were expressed as mean ± standard deviation. Skewed distribution data were expressed as median (P25, P75). Paired *t*-test compared the difference between 24 h UFC at the basal level and after DST. Chi-square test was used to assess the fundamental ratio differences among the groups. Kruskal–Wallis test was used in skewed distribution data to compare the differences among multiple groups of measurement data. The level of significance was corrected in the case of pairwise comparisons and *P*-value < 0.017 was considered statistically significant. The receiver-operating characteristic (ROC) curve was constructed to assess the usefulness of both LDDST and HDDST in the diagnosis of PPNAD. 24 h UFC (Post-L-Dex)/(Pre-L-Dex) and 24 h UFC (Post-H-Dex)/(Pre-H-Dex) were employed as test variables independently.

## Results

### Baseline characteristics and clinical features of the three groups

The baseline characteristics are presented in Table 1. A total of 25 patients with PPNAD comprised of 8 males (32%) and 17 females (68%). The BMAH group consisted of 14 females (51.9%) while the ADA group comprised of 92.9% females (78/84), suggesting that the female constituent ratio was higher in the ADA group as compared to the other two groups (*P*-value < 0.017). The mean age of the patients with PPNAD, BMAH, and ADA was 23 ± 11, 50 ± 10, and 34 ± 9 years, respectively. Although no significant difference was observed between the groups, patients with PPNAD seemed to have an earlier onset age.

**Table 1:** Baseline characteristics and clinical presentations of different groups

	PPNAD	BMAH	ADA	*P*-value
*N*	25	27	84	
Male	8 **$**	13 **#**	6	0.000
Female	17 **$**	14 **#**	78	0.000
Age of diagnosis (years)	23 ± 11	50 ± 10	34 ± 9	0.58
Hypertension	19 of 25 (76.0%)*	27 of 27 (100%) **#**	49 of 61 (80.3%)	0.03
Osteoporosis or osteopenia	18 of 23 (78.3%)	20 of 27 (74.1%)	24 of 50 (48.0%) **$**	0.015
Hypokalemia	3 of 25 (12.0%)*	14 of 27 (51.9%)	28 of 84 (33.3%)	0.009
IGT or DM	14 of 25 (56.0%)	18 of 27 (66.7%)	39 of 80 (48.8%)	0.263

*PPNAD* primary pigmented nodular adrenocortical disease, *BMAH* bilateral macronodular adrenal hyperplasia, *ADA* adrenocortical adenoma, *IGT* impaired glucose tolerance, *DM* diabetes mellitus

* PPNAD vs. BMAH, *P* < 0.017; **#** BMAH vs. ADA, *P* < 0.017; **$** PPNAD vs. ADA *P* < 0.017

As shown in Table 1, a majority of the patients exhibited typical CS manifestations such as hypertension, weight gain, and abnormal glucose metabolism, and decreased BMD. In the present study, patients in the PPNAD group seemed to have a higher incidence rate of developing decreased BMD (osteopenia or osteoporosis) than those with ADA (78.3 vs. 48.0%, *P* < 0.017), while no differences were found between PPNAD and BMAH groups. In addition, patients with PPNAD had a lower incidence rate of hypokalemia as compared to BMAH (12.0 vs. 51.9%, *P* < 0.017). In the BMAH group, all the 27 patients developed hypertension, indicating a significant difference between BMAH and other groups (100 vs. 76.0%, *P* < 0.017; 100 vs. 80.3%, *P* < 0.017). With respect to glucose metabolism, no statistically significant differences were observed among the three groups (*P* = 0.263).

### Laboratory examinations of the three groups

**Fig. 1:**
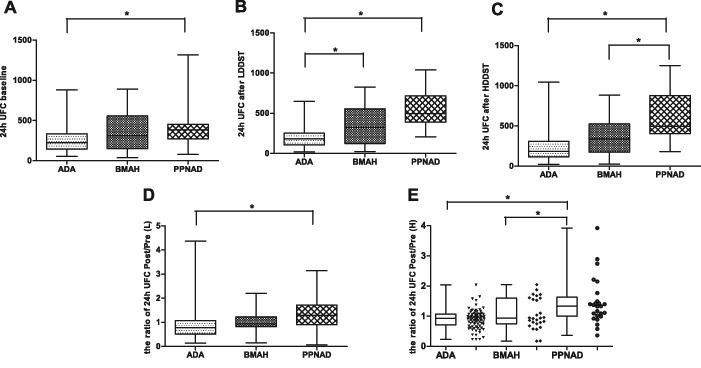
24 h UFC levels before and after administration of dexamethasone. The basal level of 24 h UFC in the PPNAD group was higher than that of ADA (Fig. 1a, *P* < 0.017), after low-dose and high-dose dexamethasone. 24 h UFC in patients with PPNAD increased, while in patients with ADA and BMAH the 24 h UFC decreased or remained unaltered. The 24 h UFC level after LDDST was higher in the PPNAD group and BMAH as compared to ADA (*P* < 0.017), while no significant difference between PPNAD and BMAH was observed (Fig. 1b). After administration of HDDST, 24 h UFC was higher in patients with PPNAD as compared to that of ADA and BMAH (Fig. 1c, *P* < 0.017). The ratio of 24 h UFC posterior to DST to before DST (Fig. 1d, e) showed a significant difference between PPNAD and the other two groups

In all participants, the serum ACTH levels were <10 pg/mL, providing support for the diagnosis of ACTH-independent CS accordingly. Table 2 displayed the 24 h UFC levels before and after administration of dexamethasone. The baseline of 24 h UFC in patients with PPNAD was higher than that of the patients with ADA (383.50 vs. 224.42, normal range: 12.3–103.5 µg/24 h, *P* < 0.017, Fig. 1a). No statistical difference was observed between the other groups. In the ADA group, the majority of the patients showed an increased UFC level, while 9.4% of the patients exhibited a 24 h UFC level within the normal range. After LDDST and HDDST, the 24 h UFC elevated in patients with PPNAD (paired *t*-test, *P* = 0.007 and *P* = 0.001); among these, 12 patients (48%) presented an elevation of 24 h UFC > 50% after HDDST. On the other hand, 24 h UFC remained unaltered in the BMAH group (paired *t*-test, *P* = 0.471 and *P* = 0.414) and decreased in the ADA group (paired *t*-test, *P* = 0.002 and *P* = 0.004, Fig. 1b, c). The 24 h UFC level after LDDST was higher in the PPNAD and BMAH groups as compared to ADA (*P* < 0.017), while no significant difference was observed between PPNAD and BMAH. After administration of HDDST, 24 h UFC was higher in patients with PPNAD as compared to that of ADA and BMAH (*P* < 0.017).

**Table 2:** Levels of 24 h UFC before and after dexamethasone

Group	PPNAD	BMAH	ADA	*P*-value
Baseline (µg/d)	383.50 (271.61, 452.97) **#**	314.70 (151.04, 557.16)	224.42 (144.50, 334.88)	0.002
LDDST (µg/d)	497.35 (389.86, 717.70) **#**	325.60 (124.28, 557.03)	181.66 (108.00, 252.00) **$**	0.000
HDDST (µg/d)	495.30 (402.97, 876.55) **#**	339.84 (175.42, 526.13) *	184.50 (116.20, 309.61)	0.000
Post/Pre (L)	1.29 (0.91, 1.71) **#**	0.93 (0.82, 1.23)	0.78 (0.51, 1.07)	0.000
Post/Pre (H)	1.48 (1.14, 2.09) **#**	0.94 (0.76, 1.59) *	0.92 (0.71, 1.07)	0.000

*PPNAD* pigmented nodular adrenocortical disease, *BMAH* bilateral macronodular adrenal hyperplasia, *ADA* adrenocortical adenoma, *LDDST* low-dose dexamethasone suppression test, *HDDST* high-dose dexamethasone suppression test, *Post/Pre* (*L*) the ratio of 24 h UFC posterior to LDDST and prior to LDDST, *Post/Pre* (*H*) the ratio of 24 h UFC posterior to HDDST and prior to HDDST

* PPNAD vs. BMAH, *P* < 0.017; **#** PPNAD vs. ADA, *P* < 0.017; $ BMAH vs. ADA, *P* < 0.017

In order to clarify the level of 24 h UFC variation, the ratio of 24 h UFC post-DST to 24 h UFC pre-DST was calculated and denoted as 24 h UFC (Post-L-Dex)/(Pre-L-Dex) and 24 h UFC (Post-H-Dex)/(Pre-H-Dex) after LDDST and HDDST, respectively. The ratio > 1 suggested that 24 h UFC elevated after DST. Furthermore, after LDDST, the 24 h UFC (Post-L-Dex)/(Pre-L-Dex) in the PPNAD group was significantly higher than that of the ADA group (1.29 vs. 0.78, *P* < 0.017), whereas the ratio between PPNAD and BMAH showed no difference (Fig. 1d). After administration of high-dose dexamethasone, 24 h UFC (Post-H-Dex)/(Pre-H-Dex) in PPNAD, BMAH, and ADA was 1.48 (1.14, 2.09), 0.94 (0.76, 1.59), and 0.92 (0.71, 1.07), respectively, and the ratio in the PPNAD group was distinctively higher than that of BMAH and ADA (*P* < 0.017, Fig. 1e).

### Clinical features and laboratory examinations between isolated PPNAD and CNC

In 9/25 patients (36.0%), PPNAD occurred as a component of CNC; the clinical features were summarized in Table 3. The cohort comprised of two males and seven females, with an average age of 19.6 (range, 14–28) years. Spotty hyperpigmentation was the most frequent manifestation and could be observed in all the patients. In addition to spotty hyperpigmentation, a 19-year-old male also demonstrated a cyst in the head of the epididymis. Multiple solid hypoechoic nodules of the thyroid gland were found in three females, whereas two had nodules in the breast. Two females had solid nodules in the unilateral or bilateral breast. No cardiac myxoma and tumors of other endocrine glands were found in patients with CNC. Furthermore, comparisons between patients with isolated PPNAD and patients with CNC (Table 4) did not reveal any significant differences in the clinical features such as age, sex, constituent ratio, body mass index (BMI), the incidence rate of hypertension, disturbance of carbohydrate metabolism, dyslipidemia, and decreased BMD. The 24 h UFC levels before and after DST did not show any difference between the patients with isolated PPNAD and those with CNC (*P* > 0.05).

**Table 3:** Clinical manifestations of the patients with CNC

Number	Gender	Age (years)	PPNAD	Other manifestations of CNC
1	M	14	Yes	Spotty hyperpigmentation in lower eyelid and lips
2	F	23	Yes	Spotty hyperpigmentation in face and bulbar conjunctiva
3	F	17	Yes	Spotty hyperpigmentation in face and hands, with similar family history
4	F	28	Yes	Spotty hyperpigmentation in lips and buccal mucosa, pancreatic cyst, nodule in unilateral breast
5	F	19	Yes	Spotty hyperpigmentation in face and lips, solid nodules in bilateral breast
6	F	14	Yes	Multiple hypoechoic nodules of the thyroid gland
7	M	19	Yes	Spotty hyperpigmentation in face, cyst in the head of epididymis
8	F	25	Yes	Spotty hyperpigmentation in lips and buccal mucosa, hypoechoic nodules in thyroid gland and breast, skeletal lesions
9	F	17	Yes	Spotty hyperpigmentation in lips and buccal mucosa, hypoechoic nodules in thyroid gland and breast

*PPNAD* pigmented nodular adrenocortical disease

**Table 4:** Clinical features and laboratory examinations of patients with isolated PPNAD and patients with CNC

	Isolated PPNAD	Carney syndrome	*P*-value
Age (years)	22.13 ± 9.91	19.56 ± 4.57	0.318
Number	16	9	0.667
Female	10	7	
Male	6	2	
BMI (kg/m^2^)	24.85	26.63	0.327
Hypertension	11	8	0.380
Grade I	3	2	
Grade II	2	3	
Grade III	6	3	
IGT or DM	9	5	1.000
IGT	8	4	
DM	1	1	
TC (mmol/L)	6.01	5.25	0.213
TG (mmol/L)	1.81	1.46	0.491
LDL-C (mmol/L)	3.81	3.03	0.122
HDL-C (mmol/L)	1.65	1.61	0.869
Potassium (mmol/L)	4.00	3.96	0.873
Osteopenia or osteoporosis	12	6	1.000
Osteopenia	3	1	
Osteoporosis	9	5	
Severe osteoporosis	2	1	
Fatty liver	5	3	1.000
Adrenal gland CT	4 with normal adrenal glands	2 with normal adrenal glands	
	5 with unilateral nodules	1 with unilateral nodules	
	8 with bilateral nodules	6 with bilateral nodules	
24 h UFC			
Baseline	375.05 (282.18, 411.40)	423.10 (216.88, 622.96)	0.301
LDDST	482.20 (344.96, 770.60)	556.92 (389.86, 778.85)	0.558
HDDST	485.42 (339.17, 745.01)	862.90 (391.21, 948.76)	0.229
Post/Pre (L)	1.31 (1.04, 1, 75)	1.50 (0.79, 2.15)	0.978
Post/Pre (H)	1.49 (1.12, 2.08)	1.59 (1.05, 2.18)	0.846

*PPNAD* pigmented nodular adrenocortical disease, *BMI* body mass index, *IGT* impaired glucose tolerance, *DM* diabetes mellitus, *TC* total cholesterol, *TG* triglyceride, *LDL-C* low-density lipoprotein cholesterol, *HDL-C* high-density lipoprotein cholesterol, *24 h UFC* 24 h urinary free cortisol, *Post/Pre* (*L*) the ratio of 24 h UFC posterior to LDDST and prior to LDDST, *Post/Pre* (*H*) the ratio of 24 h UFC posterior to HDDST and prior to HDDS

### ROC and cut-off values in the diagnosis of PPNAD

**Fig. 2:**
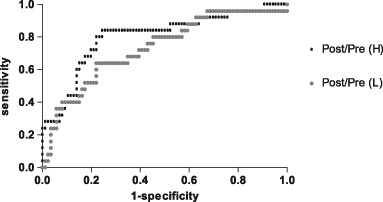
ROC curve was constructed with the ratio of 24 h UFC Post/Pre (L) and Post/Pre (H) as the detection variable. The AUC was 0.733 and 0.802 after low-dose and high-dose dexamethasone, respectively

24 h UFC (Post-L-Dex)/(Pre-L-Dex) and 24 h UFC (Post-H-Dex)/(Pre-H-Dex) were used as the test variables. The ROC curve was constructed with PPNAD that was served as the state variable, and its value was 1. Taking the leftmost point of the curve as the cut-off point, the area under the ROC curve (AUC) was 0.733 after LDDST, and the cut-off value of 24 h UFC (Post-L-Dex)/(Pre-L-Dex) was 1.16 with 64.0% sensitivity and 77.9% specificity. Similarly, the AUC was 0.802 after HDDST, and the cut-off value of 24 h UFC (Post-H-Dex)/(Pre-H-Dex) was 1.08 with 84.0% sensitivity and 75.6% specificity (Fig. 2).

## Discussion

PPNAD occurs as a rare cause of CS, accounting for 0.6–1.9% of all such patients [2]. CNC is an autosomal dominant inherited and multiple neoplasia syndrome characterized by skin tumors and pigmented lesions, cardiac myxomas, schwannomas, breast adenomas, bone lesions and various endocrine disorders caused by tumors of the pituitary and thyroid glands, pancreas, and/or gonads [10, 11]. A total of 26–60% of the CNC patients exhibited PPNAD, and it is the most common endocrine tumor associated with CNC [3, 10, 12]. In our study, 36.0% PPNAD patients had the components of CNC, and all of them presented spotty pigmentation in the skin or the mucosa, five females demonstrated the hypoechoic nodules of the thyroid gland and/or breast, and a male developed a cyst in the head of the epididymis. Nevertheless, the commonly reported manifestation, cardiac myxoma [13], was not found in our patients. Stratakis et al. reported that 20/21 patients (95%) showed PPNAD that occurred as a component of CNC [4]. Such an enormous difference in the incidence rate of CNC in patients with PPNAD between the current study (36%) and that by Stratakis et al. (95%) might be attributed to the ethnic differences. Additionally, the sample size of both studies was not sufficiently large, which leads to inevitable bias to the results. Further investigations with expanded sample size need to be undertaken in order to clarify the genetic distinctions among various races.

DST is commonly used in the diagnosis and differential diagnosis of ACTH-dependent CS. LDDST or 1 mg overnight DST is used for screening the presence of CS in the patient, while HDDST is used to distinguish CS from other ACTH-dependent CS; >50% suppression of cortisol concentration indicates CS. However, limited studies are available concerning the administration of dexamethasone in ACTH-independent CS since the baseline of serum ACTH level is relatively low. Several reports have mentioned a paradoxical increase in the glucocorticoid level after administration of dexamethasone in patients with PPNAD [14–17]. Silverman et al. reported a patient with CS secondary to bilateral nodular adrenocortical hyperplasia, in whom urinary 17-OHCS could not be suppressed by a large dose of dexamethasone, and the adrenal pathology demonstrated cortical hyperplasia with multiple small nodules [14]. In 1999 Stratakis et al. found that UFC levels on day 6 of the Liddle test had the highest accuracy in the diagnosis of PPNAD, and an increase of >50% UFC levels could discriminate 9/13 patients with PPNAD from other primary adrenocortical disorders [4]. In the current study, we also found that after DST, the 24 h UFC levels in patients with PPNAD increased, while in the other two groups the 24 h UFC levels remained unchanged or even decreased, which suggested the usefulness of DST in the diagnosis of PPNAD. In patients with ADA, the 24 h UFC levels were slightly decreased after DST, which might be partially attributed to the suppressibility of those patients whose basal 24 h UFC values were within the normal range.

Herein, we used the ratio of 24 h UFC post-dexamethasone to prior-dexamethasone, for the first time, in the diagnosis of PPNAD. After LDDST, the ratio of 24 h UFC Post/Pre (L) in the PPNAD group was significantly higher than that in the ADA group, while it could not differentiate PPNAD from BMAH. After administration of high-dose dexamethasone, a statistically significant difference between PPNAD and BMAH was noted, indicating the diagnostic value of DST, especially the HDDST could identify PPNAD from both BMAH and ADA. Our study revealed the ratio of 24 h UFC after LDDST to the baseline value >1.16 with 64.0% sensitivity and 77.9% specificity. Similarly, when the value of 24 h UFC (Post-H-Dex)/UFC (Pre-H-Dex) is >1.08, the sensitivity and specificity increased to 84.0 and 75.6%, respectively. Compared to the previous study using 24 h UFC of >50% increase on day 6 of the Liddle test, the degree of UFC level was slightly lower in our study. This phenomenon might be attributed to the theory that the proportion of patients with isolated PPNAD was different between our study and that by Stratakis et al. The molecular mechanism underlying the paradoxical response to dexamethasone in patients with PPNAD was due to an increased expression of glucocorticoid receptor in PPNAD nodules [17]. The CNC is primarily caused by germline mutations in the protein kinase A regulatory subunit 1A (*PRKAR1A*) gene [18–22]. Nevertheless, several studies demonstrated that patients with isolated PPNAD exhibited specific molecular genetic abnormalities with mutations in phosphodiesterase *PDE11A*, *PDE8B*, and the PKA catalytic subunit *PRKACA* gene [23–25]. The mutations in various gene loci may differentially influence the cAMP/PKA pathway in patients with isolated PPNAD and CNC, leading to variable levels of UFC.

Nevertheless, the present study exhibited some limitations. Since we mainly focused on the clinical study regarding the usefulness of DST in the diagnosis of PPNAD, the genetic mutations were not assessed in the participants, which made it impossible to compare the genetic abnormities between patients with isolated PPNAD and those with CNC. Furthermore, due to the retrospective nature of the current study, some clinical data were missing, which could lead to a potential bias in our study.

In conclusion, DST, especially HDDST, is greatly beneficial in the early diagnosis of PPNAD. When the radiological examination could not provide valuable information, DST could detect the patients with subclinical, atypical PPNAD-associated manifestations and distinguish PPNAD from other adrenal diseases. With respect to the Chinese population, the cut-off value of 24 h UFC (Post-H-Dex)/(Pre-H-Dex) was 1.08 with 84.0% sensitivity and 7.6% specificity. However, the cut-off values of 24 h UFC levels between different races need to be investigated further.
